# Associations between frontal lobe activity and depressive symptoms in patients with major depressive disorder receiving rTMS treatment: a near-infrared spectroscopy study

**DOI:** 10.3389/fpsyt.2023.1235713

**Published:** 2023-08-16

**Authors:** Po-Han Chou, Wen-Chun Liu, Shao-Cheng Wang, Wei-Hao Lin, Yi-Lun Chung, Chun-Hung Chang, Kuan-Pin Su

**Affiliations:** ^1^Department of Psychiatry, Hsinchu Hospital, China Medical University, Hsinchu, Taiwan; ^2^An-Nan Hospital, China Medical University, Tainan, Taiwan; ^3^Department of Psychiatry, Taoyuan General Hospital, Ministry of Health and Welfare, Taoyuan, Taiwan,; ^4^Department of Mental Health, Johns Hopkins Bloomberg School of Public Health, Baltimore, MD, United States; ^5^Department of Medical Laboratory Science and Biotechnology, Chung Hwa University of Medical Technology, Tainan, Taiwan; ^6^Department of Psychiatry, Puli Branch, Taichung Veterans General Hospital, Taichung, Taiwan; ^7^Institute of Medicine, Chung Shan Medical University, Taichung, Taiwan; ^8^Mind-Body Interface Research Center (MBI-Lab), China Medical University Hospital, Taichung, Taiwan; ^9^College of Medicine, China Medical University, Taichung, Taiwan

**Keywords:** depressive symptom, frontal lobe activity, major depressive disorder, near infrared spectroscopy, NIRS, repetitive transcranial magnetic stimulation, rTMS

## Abstract

**Introduction:**

The effects of repetitive transcranial magnetic stimulation (rTMS) on the left dorsolateral prefrontal cortex (DLPFC) in patients with major depressive disorder (MDD) have been proved to have antidepressant effects. However, the absence of biomarkers to assess treatment response remains a challenge. This research aims to explore the relationship between frontal lobe activity, measured using near infrared spectroscopy (NIRS), and changes in symptoms among MDD patients following rTMS treatment.

**Methods:**

A total of 26 MDD patients underwent 20 sessions of 10  Hz rTMS targeting the left DLPFC. NIRS was used to measure frontal lobe activity during a verbal fluency test at baseline, after 10 rTMS sessions, and after 20 rTMS sessions. Responders were defined as individuals with more than a 50% reduction in symptoms based on the 21-item Hamilton Depression Rating Scale after 20 rTMS sessions.

**Results:**

Among the 14 responders, an increase in frontal lobe activity was significantly correlated with improvements in depressive symptoms following 10 (*p* = 0.0001) and 20 rTMS sessions (*p* = 0.007). Additionally, frontal lobe activity after 10 rTMS sessions was significantly associated with symptom improvement after 20 sessions (*p* = 0.001). These associations were not observed among non-responders.

**Conclusion:**

The findings from this study indicate distinct patterns of frontal lobe activity between responders and non-responders to rTMS treatment, suggesting that NIRS has the potential to serve as a biomarker for monitoring treatment response in MDD patients undergoing rTMS.

## 1. Introduction

The U.S. Food and Drug Administration has authorized the use of repetitive transcranial magnetic stimulation (rTMS) and intermittent theta burst stimulation (iTBS) as treatments for depression that does not respond to standard medication. Extensive evidence supports the effectiveness of high-frequency rTMS or iTBS, particularly when targeted at the left dorsolateral prefrontal cortex (DLPFC), in producing significant antidepressant effects (1). However, the response rate to these therapies remains imperfect, as evidenced by recent randomized control trials reporting a response rate of less than 50% (2). One of the primary factors contributing to this variability is the distinct response trajectories exhibited by patients with major depressive disorder (MDD) undergoing rTMS treatment (3). In a study conducted by Kaster et al., four response trajectories were identified among 388 MDD patients receiving rTMS/iTBS: nonresponse (*N* = 43, 11%), rapid response (*N* = 73, 19%), linear response with higher baseline symptoms (*N* = 118, 30%), and linear response with lower baseline symptoms (*N* = 154, 40%). Notably, significant differences in response rates between these trajectories were observable as early as the first week of treatment (3). These findings underscore the necessity for personalized rTMS treatment plans and the identification of biomarkers for assessing clinical outcomes in patients with MDD (1).

The progress made in neuroimaging methods has underscored the significance of biomarkers in neuroimaging studies, offering the potential for precision medicine in the treatment of major depressive disorder (MDD). Various neuroimaging techniques are being employed to investigate factors influencing treatment response. These approaches encompass brain volumetric MRI, functional MRI (fMRI) including resting-state and affective tasks, electroencephalography (EEG), diffusion tensor imaging (DTI), near-infrared spectroscopy (NIRS), as well as molecular imaging techniques such as positron emission tomography (PET) and single photon emission computed tomography (SPECT). These diverse neuroimaging tools are being harnessed to explore and identify potential modulators of treatment response in MDD, thereby facilitating the development of personalized treatment approaches (1). Imaging techniques have been utilized to forecast treatment response or recurrence in individuals with major depressive disorder (MDD), depending on the specific objectives and nature of the studies conducted. For instance, an Australian study employed functional MRI (fMRI) analysis on a sample of 26 patients with treatment-resistant MDD. These individuals underwent a planning task both before and after receiving rTMS treatment. The study findings revealed that patients who positively responded to low-frequency rTMS treatment exhibited decreased bilateral activation of the middle frontal gyrus. This highlights the potential of fMRI analysis to predict treatment outcomes in MDD patients and underscores the importance of such imaging techniques in understanding the effects of therapeutic interventions (4). Baeken et al. conducted a study in which they observed that elevated baseline glucose metabolism in the anterior cingulate cortex, assessed through positron emission tomography (PET) scans using 18F-fluoro-2-deoxy-D-glucose radiotracer (18FDG-PET), served as a marker indicating a favorable response to repetitive transcranial magnetic stimulation (rTMS) (5). SPECT studies have been employed to predict treatment response in individuals with major depressive disorder (MDD), encompassing both pharmacotherapy and nonpharmacologic therapies like psychotherapy. Notably, SPECT-guided repetitive transcranial magnetic stimulation (rTMS) has emerged as a valuable approach for guiding the selection of brain stimulation regions in the treatment of medication-resistant MDD. The stimulation of hypoperfused regions in the left prefrontal cortex using rTMS has demonstrated superior improvements compared to targeting the left dorsolateral prefrontal cortex. This highlights the potential of SPECT-guided rTMS as a valuable tool for decision-making in the application of brain stimulation techniques for individuals with treatment-resistant MDD (6). In a study focusing on proton magnetic resonance spectroscopy (MRS) in individuals with treatment-resistant major depressive disorder (MDD), researchers examined the prefrontal cortex before and after repetitive transcranial magnetic stimulation (rTMS). The findings of this study revealed a notable positive correlation between changes in myo-inositol metabolism and clinical improvements in depression, underscoring the significance of myo-inositol as a potential marker for assessing treatment response in MDD (7).

NIRS, an advanced functional neuroimaging technology, enables the non-invasive assessment of the spatial and temporal patterns of neural activity in frontotemporal regions (8). NIRS provides several advantages over conventional imaging techniques like PET, SPECT, and fMRI. NIRS offers several advantages over conventional imaging techniques such as PET, SPECT, and fMRI. Notably, unlike fMRI assessments, which require individuals to be positioned uncomfortably in a narrow gantry with their head fixed throughout the examination, NIRS measurements can be obtained in ordinary clinical settings, allowing patients to be comfortably seated in a well-lit room.

Similar to PET and SPECT, which are capable of detecting changes in cerebral blood flows, NIRS exhibits superior time-resolution with a sampling time of 0.1 s and moderate spatial resolution (9). On the other hand, EEG, the widely used electrophysiological method in various research areas, including rTMS treatment in patients with MDD (1), offers excellent temporal resolution within the millisecond range but suffers from poor spatial resolution due to volume conduction, limiting its localization capabilities (10). In comparison, NIRS provides good spatial resolution but lower temporal resolution due to the inherent hemodynamic delay (11). Furthermore, NIRS demonstrates greater resilience to electrical noise and motion-based muscle activity artifacts compared to EEG (12). NIRS measures oxy-hemoglobin [(oxy-Hb)] and deoxy-hemoglobin [(deoxy-Hb)] concentrations at the bedside. These measurements are believed to reflect regional cerebral blood volumes and exhibit robust correlations with fMRI signals (13). There is growing evidence that NIRS can aid in diagnosing and predicting treatment response in MDD (14). Previous studies have demonstrated increased frontal lobe activity measured by NIRS in patients with MDD receiving rTMS treatment. However, the associations between changes in frontal lobe activity and clinical symptoms remain unknown. Furthermore, to our knowledge, changes in frontal lobe activity between responders and non-responders have never been investigated. Therefore, this study has two primary objectives: firstly, to compare the frontal lobe activity during a verbal fluency test (VFT) between individuals who respond to rTMS treatment and those who do not; and secondly, to examine the relationships between frontal lobe activity and changes in symptoms among patients undergoing rTMS therapy.

## 2. Materials and methods

### 2.1. Study subjects

This study included 26 patients with MDD who received 20 sessions of rTMS treatment between August 2012 and July 2017 at the Department of Psychiatry, Taichung Veterans General Hospital, Taichung City, Taiwan. There were 14 patients who responded to rTMS treatment (responder) and 12 patients who did not respond (non-responder). No significant differences regarding distributions of age, gender, educational level and baseline frontal activity were noted. Similar to many previous studies, responders were defined as participants who achieved a reduction of ≥50% in the 17-item Hamilton Depression Rating Scale (HAMD-17) (15) scores after rTMS treatment. Based on previous studies, we chose 20 sessions of rTMS treatment to evaluate the effectiveness (3, 16, 17).

To be included in the study, participants needed to meet the following inclusion criteria: (1) meet the DSM-IV diagnostic criteria for major depressive disorder (MDD), validated using the Mini International Neuropsychiatric Interview (MINI) (18); (2) be between 18 and 65 years old; (3) have experienced treatment failure with at least one antidepressant or demonstrated significant intolerance to antidepressant medications; (4) possess good physical health, as confirmed by medical history and physical examination.

Patients were excluded from the study if they met any of the following exclusion criteria: (1) presence of comorbid Axis I disorders (except nicotine dependence), including psychotic disorders, bipolar disorders, or organic mental disorders; (2) current pregnancy; (3) history of seizure disorder; (4) history of neurological disorders; (5) presence of ferromagnetic material near or in the body (such as implanted pacemaker or medication pump, metal plate in the skull, or metal objects in the eyes or skull), or individuals with cochlear implants. The study was conducted following the principles of the Declaration of Helsinki, and all participants provided written informed consent. The study protocol received approval from the Institutional Review Board of the Taichung Veterans General Hospital (approval no. CF13044).

### 2.2. TMS treatment

All patients underwent a total of 20 sessions of 10 Hz rTMS targeting the left-sided dorsolateral prefrontal cortex (DLPFC) at Taichung Veterans General Hospital. The rTMS was administered using a MAGSTIM 200 and figure-of-eight coils (Magstim Co., Whitland, Dyfed, United Kingdom). Patients received 5 sessions per week. The stimulation intensity utilized in the study was set at 80% of the resting motor threshold (RMT). The RMT was measured by stimulating the right first dorsal interosseous muscle using a hand-held figure-of-eight coil (18). The DLPFC was located using Beam F3 method (19).

### 2.3. Clinical assessment

**Figure 1:**
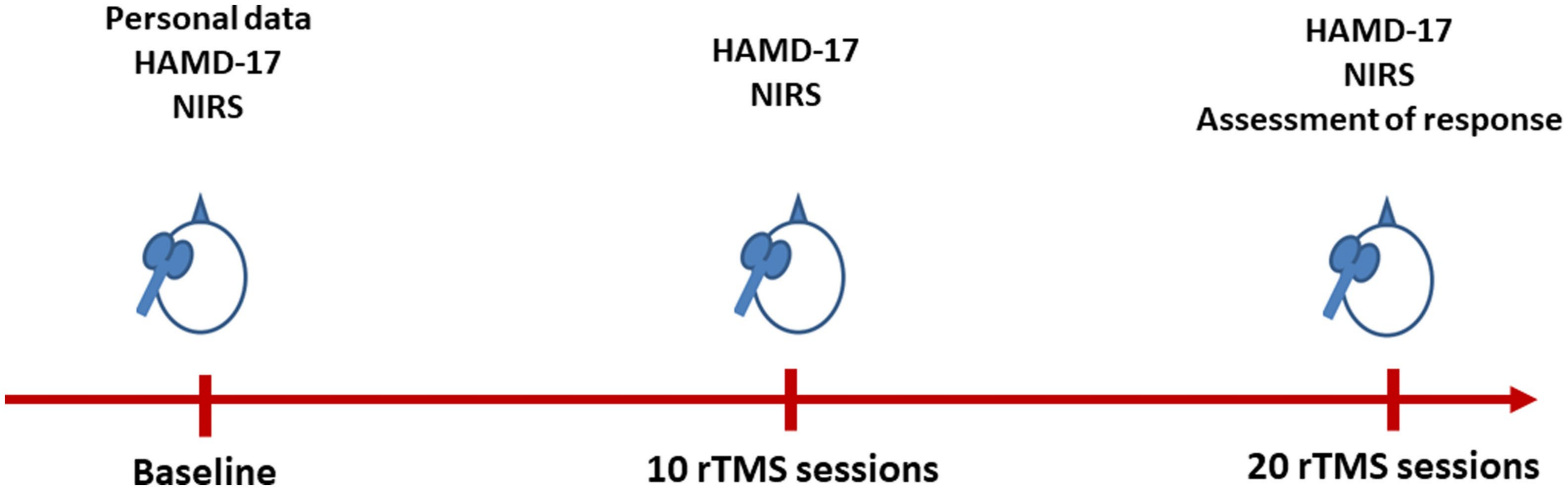
Flow chart of the present study. HAMD-17, 17-item Hamilton Depression Rating Scale; NIRS, near-infrared spectroscopy; rTMS, repetitive transcranial magnetic stimulation.

Patient characteristics, including age, sex, years of education, and concomitant medication usage, were collected. Refractoriness was assessed using the Maudsley staging method (MSM) (20). The changes in depressive symptoms, as evaluated by HAMD-17 scores at three time points: baseline (T0), after 10 sessions (T1), and after 20 sessions (T2) of rTMS treatment were also assessed (Figure 1).

### 2.4. Verbal fluency test

The patients underwent 125-s block-design verbal fluency tasks (VFTs), a well-established and commonly employed paradigm in numerous previous fNIRS studies (21–24). The verbal fluency task (VFT) comprised three distinct time periods: a 10-s pre-task period, a 60-s task period, and a 55-s post-task period. During the pre- and post-task periods, patients were instructed to engage in repetitive counting from one to five to account for and eliminate motion artifacts associated with the task. Within the 60-s task period, participants were instructed to generate words beginning with a specific phonological syllable presented by the NIRS machine. The VFT included three consecutive 20-s sub-periods, each initiated by a single Chinese syllable selected from nine available options. These options included the syllables /ㄅ(b)/, /ㄆ(p)/, or /ㄉ(d)/ for the first sub-period, /ㄊ(t)/, /ㄌ(l)/, or /ㄋ(n)/ for the second sub-period, and /ㄇ(m)/, /ㄈ(f)/, or /ㄘ(dz)/ for the third sub-period. Prior to each task session, participants received instructions on generating correct responses for the VFT.

### 2.5. NIRS measurement

**Figure 2:**
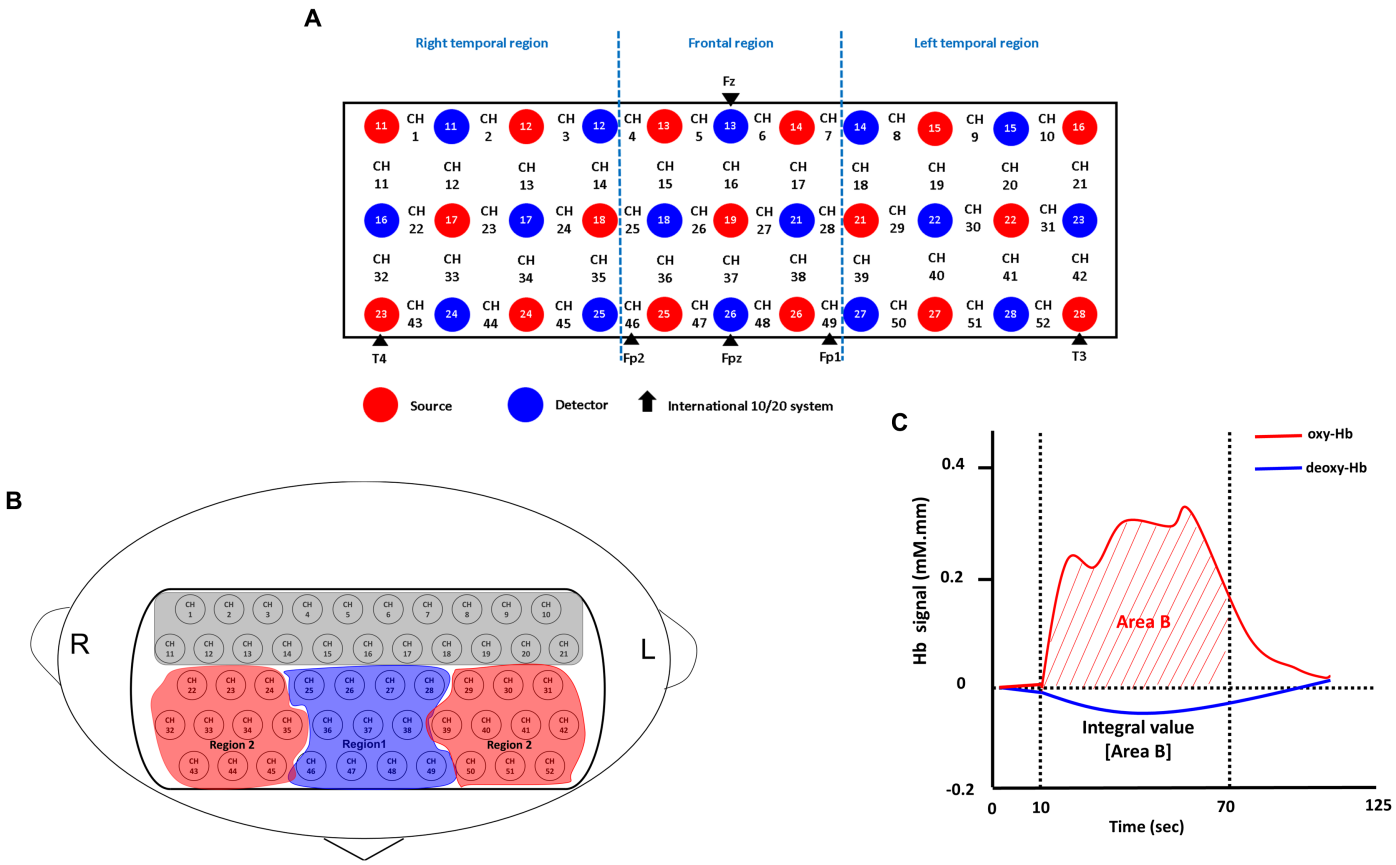
Probe setting and measurement points for the 52-channel NIRS. **(A)** The localizations of channels was determined based on the international 10–20 electroencephalography system. Red and blue circles, respectively indicate the emitter and detector of the near-infrared light. **(B)** Two regions of interest (Regions 1 and 2) in the present study. Region 1 consists of 11 channels (ch 25–28, ch 36–38 and ch 46–49); Region 2 consists of 20 channels; Right: (ch 22–24, ch 32–35 and ch 43–45); Left: (ch 29–31, ch 39–42 and ch 50–52). **(C)** Typical time-course pattern of NIRS signals during the VFT. The “integral value (Area B)” indicated the brain activity during the test; Oxy-Hb, oxygenated hemoglobin; deoxy-Hb, deoxygenated hemoglobin.

In this study, brain oxygenated hemoglobin [oxy-Hb] concentrations were measured using a 52-channel NIRS instrument (ETG-4000; Hitachi Medical Co., Tokyo, Japan). The NIRS probe attachments consisted of thermoplastic shells arranged in a 3 × 11 configuration, totaling 52 channels (Figure 2). The placement of the probes followed the Fp1–Fp2 line, in accordance with the international 10–20 system commonly used in EEG. The NIRS instrument employed two near-infrared light wavelengths (695 nm and 830 nm) to measure changes in both [oxy-Hb] and [deoxy-Hb] (in mM). The calculations were based on the Beer–Lambert law (25). Changes in [oxy-Hb] from the baseline to the activation period were measured, and relative changes were evaluated in mM·mm units. The NIRS instrument utilized an integral mode with a data sampling rate of 0.1 s. A moving average technique with a window width of 5 s was implemented, and the machine automatically detected and discarded any motion artifacts (26). The estimation of spatial information for each channel was performed using data obtained from the Functional Brain Science Laboratory at Chuo University in Japan (27) utilizing the LONI Probabilistic Brain Atlas (LPBA40) (28).

The indicator of frontal lobe activity during the verbal fluency task (VFT) was determined based on the integral value of the bilateral frontotemporal regions, as depicted in Figure 1. Further details on the definition of the integral value can be found in previous works (26, 29). In summary, the integral value represents the magnitude of the hemodynamic response throughout the 60-s test period (26). Due to the limited sample size in this study, conducting channel-wise analyses with correction for multiple comparisons (e.g., false discovery rate) might be excessively stringent for detecting differences. Instead, following the approach of previous studies (23, 26–28), we focused on a specific region of interest, as depicted in Figure 1, namely the frontal region (R1), which consists of 11 channels. Based on the LBPA40 (28), the NIRS signal from “Region 1 (R1)” encompassed signals from channels located in the fronto-polar and dorsolateral prefrontal cortical regions. To assess brain activity, we focused on changes in [oxy-Hb] over the bilateral frontal region (R1) during the VFT, as it has demonstrated stronger correlations with fMRI blood-oxygenation level-dependent signals compared to [deoxy-Hb] (26).

### 2.6. Statistical analysis

Demographic and clinical data were analyzed using STATA 15 (StataCorp, College Station, TX, United States). The distribution of the data was examined using the Kolmogorov–Smirnov or Shapiro–Wilk tests. In cases where the data violated the assumptions for parametric testing, non-parametric analysis methods such as the Mann–Whitney U and Wilcoxon signed-rank sum tests were employed to compare differences. Alternatively, independent and paired *t*-tests were used when the data met the assumptions for parametric testing. For categorical variables with small sample sizes, Fisher’s exact or chi-square tests were utilized for analysis. To explore the associations between changes in depressive symptoms and brain activities during the VFT in the responder and non-responder groups, Pearson’s correlation or Spearman’s rho was employed. Furthermore, one-way repeated measures analysis of variance (ANOVA) was conducted to examine the longitudinal changes in frontal activity between T0, T1 and T2 in both groups. A receiver operating characteristic (ROC) analysis was performed and used to generate simple indices of NIRS signal patterns, to aid individual prediction. Effect size was indicated with Cohen’s d. To elucidate the associations between baseline frontal activity and treatment response, logistic regression was performed with age, sex, education, MSM scores, previous episodes, baseline HAMD-17 scores and baseline frontal activity as independent factors; responders after 20 sessions (≥ 50% reduction in the HAMD-17 scores) were the dependent factors. Statistical significance was set at *p* < 0.05 for two-sided tests.

## 3. Results

### 3.1. Basic characteristics

Twenty-six subjects (14 responders and 12 non-responders) were included in the analysis. Baseline demographic and clinical characteristics (i.e., age, gender, education, MSM scores, HAMD-17 scores, and frontal lobe activity at baseline and medication use) were similar between the two groups (Table 1).

**Table 1:** Characteristics of study participants.

	Responders (*n* = 14)	Non-responders (*n* = 12)	*p-*value
Characteristics			
Age (y)	60.0 (8.5)	60.9 (7.9)	0.78
Sex (male/female)	6/8	5/7	0.95
Education years (y)	14.8 (3.0)	15.5 (3.2)	0.56
MSM	3.36 (0.74)	3.50 (0.67)	0.62
VFT performance	13.8 (3.20)	14.8 (3.44)	0.50
Previous episodes	3.43 (1.87)	3.17 (1.52)	0.70
HAMD-17 measurements			
Baseline	36.5 (11.07)	38.3 (9.94)	0.68
10 rTMS sessions	25.4 (7.39)	31 (8.92)	0.09
20 rTMS sessions	12.9 (4.48)	25.2 (8.04)	**0.001**
HAMD changes after 10 sessions	−11.1 (6.04)	−7.5 (3.84)	0.069
HAMD change after 20 sessions	−23.6 (9.9)	−13.0 (5.05)	**0.003**
Frontal function assessment measures (Integral value of R1)			
Integral value changes after 10 sessions	16.4 (11.5)	- 9.6 (10.99)	**< 0.001**
Integral value changes after 20 sessions	54.8 (32.0)	11.3 (9.29)	**0.001**
Integral value at baseline	73.2 (15.8)	64.4 (12.0)	0.13
Medications			
Antidepressant (One/two/three)	6/6/2	6/6/0	0.395
BZD	13	12	0.345
Mood stabilizer	5	3	0.555
Antipsychotics	6	5	0.951

Means (standard deviation); MSM, Maudsley staging method; VFT, verbal fluency test; rTMS, repetitive transcranial magnetic stimulation; BZD, benzodiazepine; HAMD, Hamilton Depression Rating Scale. Bold values indicated *p* < 0.05.

### 3.2. Changes in frontal lobe activity after rTMS treatment

**Figure 3:**
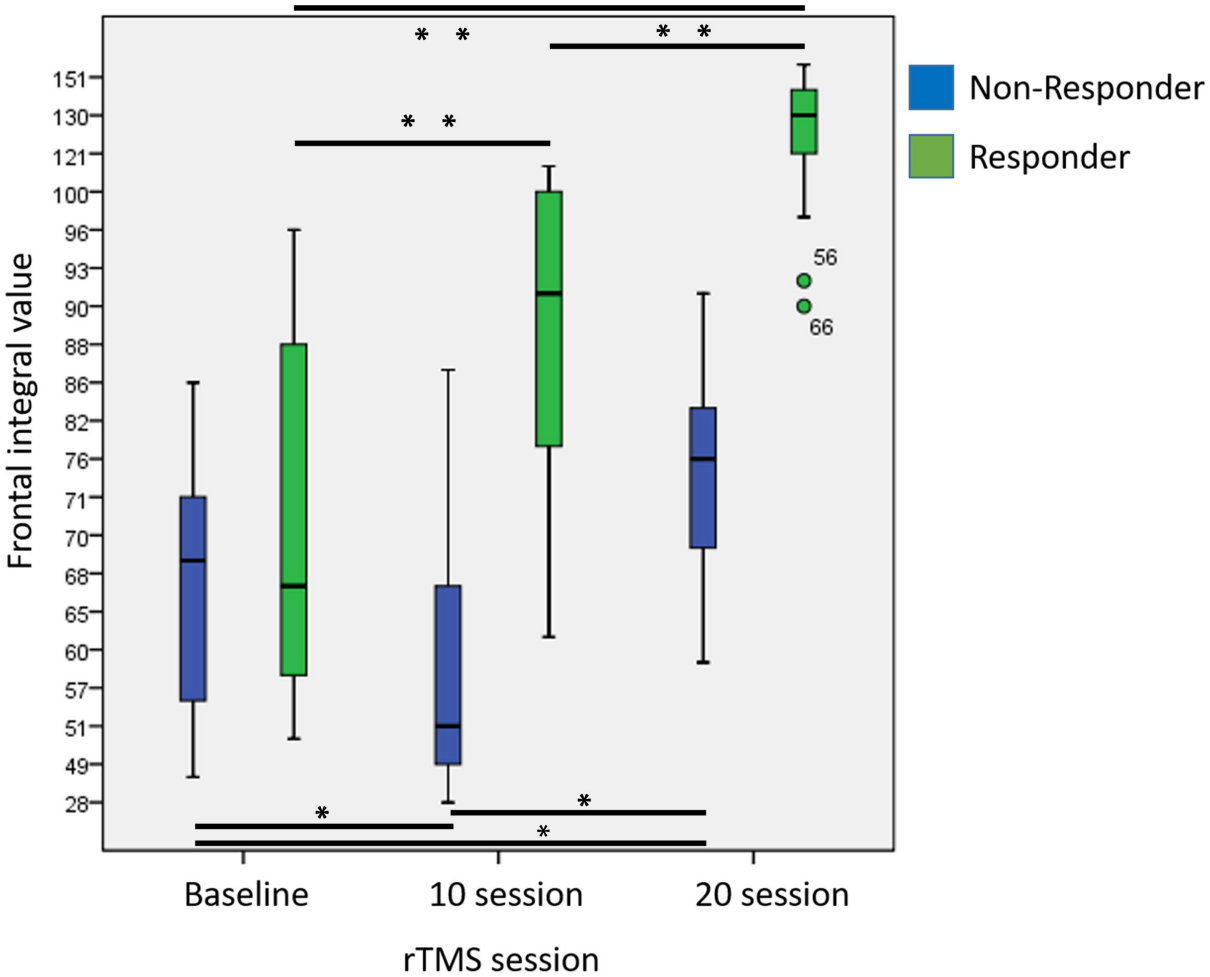
Changes in frontal integral value at baseline, after 10 and 20 sessions in responders and non-responders. (Asterisk indicated results of repeated measures of ANOVA for comparisons of frontal integral values at different time point; *p* < 0.05^*^, *p* < 0.001^**^).

Compared to baseline (T0), the responder group showed significantly increased frontal lobe activity at T1 (*p* < 0.001) and T2 (*p* < 0.001) respectively. In addition, frontal activity also significantly increased at T2 compared to T1 (*p* < 0.001). In the non-responder group, compared to the frontal activity at T0, there was a significant decrease at T1 (*p* = 0.012) but a significant increase at T2 (*p* = 0.001). Compared to T1, there was also a significant increase in frontal activity at T2 (*p* = 0.001) (Figure 3).

Moreover, the responder group demonstrated significantly greater increases in frontal lobe activity compared to non-responder group both at T1 (*p* < 0.001; Cohen’s *d* = 2.30) and T2 (*p* = 0.001; Cohen’s *d* = 1.85).

### 3.3. Associations between changes in frontal lobe activity during VFT and depressive symptoms

**Figure 4:**
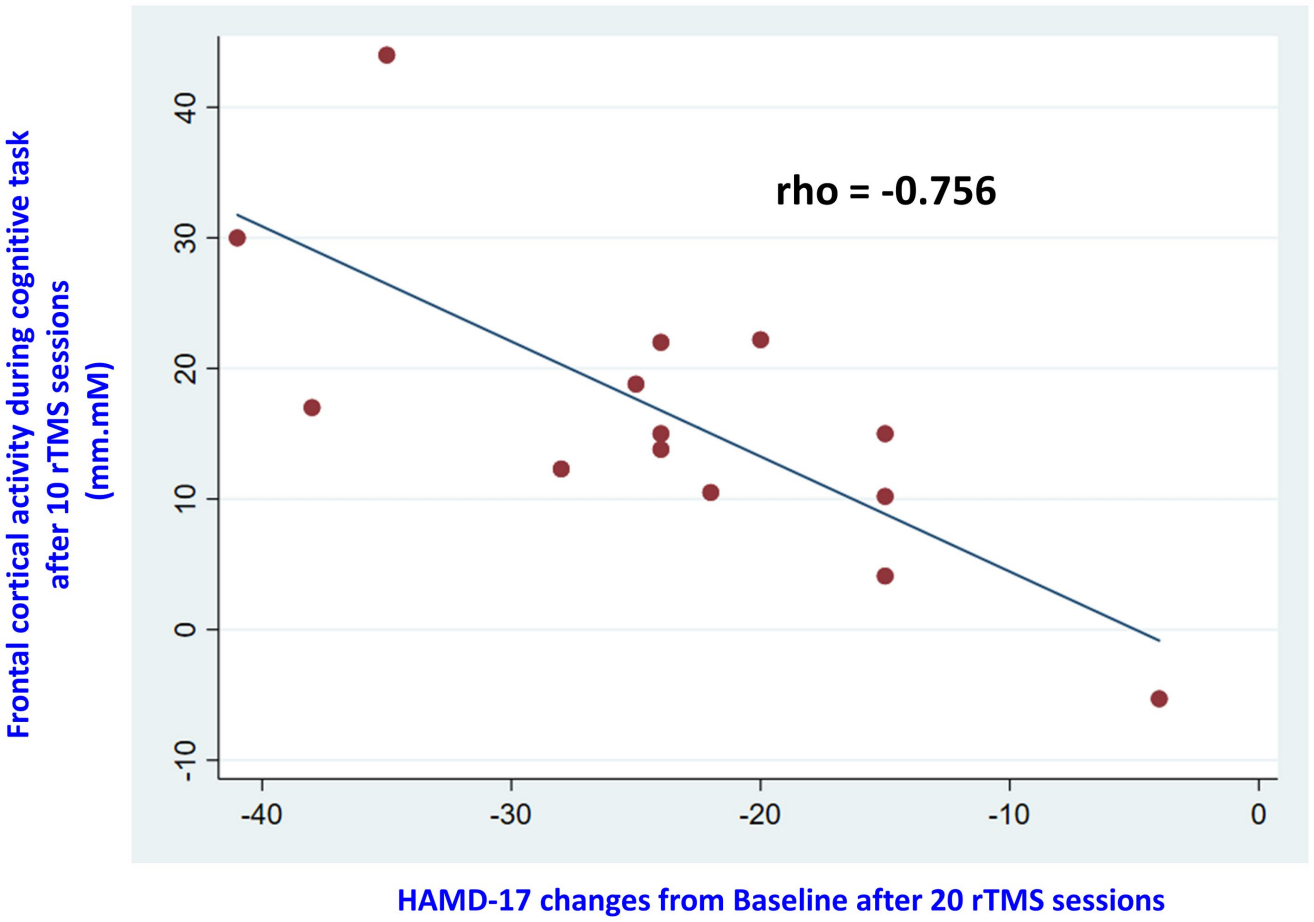
The scatterplot displayed correlations between changes in frontal lobe activity after 10 sessions of rTMS and HAMD-21 scores after 20 sessions among responders.

At the baseline, there were significant negative correlations between frontal.

lobe activity and depressive symptoms in all 26 subjects (rho = −0.693, *p* = 0.001). The results of logistic regression revealed that baseline HAMD-17 scores and baseline frontal activity were not associated with treatment responses. Among the responders, there was a significant association between increased frontal lobe activity and improvement in depressive symptoms after 10 rTMS treatments (rho = −0.868, *p* = 0.0001) and 20 rTMS treatments (rho = −0.682, *p* = 0.007). However, this association was not observed in the non-responders. Moreover, among the responders, increased frontal lobe activity after 10 sessions of rTMS was significantly associated with an improvement in depressive symptoms after 20 sessions (rho = −0.756, *p* = 0.001) (Figure 4). In contrast, this association was not observed in the non-responders (rho = −0.153, *p* = 0.634). Using increases in R1 with 2.8 [mm.Mm] at T1 as the optimal threshold, the resulting area under the ROC curve value was 0.97 [95% CI, (0.00–1.00); sensitivity: 0.93, specificity: 1.00].

## 4. Discussions

To the best of our knowledge, this is the first study to investigate the associations between changes in frontal lobe activity measured by a 52-channel NIRS and improvements in depressive symptoms in MDD patients who either responded or did not respond to rTMS treatment. In the present study, we found increased frontal lobe activity was associated with improved depressive symptoms in rTMS responders. Additionally, increased frontal lobe activity after 10 sessions of rTMS was found to be associated with improved symptoms after 20 sessions of rTMS treatment. However, those associations were not observed among non-responders.

Consistent with previous NIRS studies [reviewed by Ho et al. (14)], we observed significant negative associations between depressive symptoms and frontal lobe activity during the VFT in patients with MDD. On the other hand, few studies have investigated the effects of rTMS on frontal lobe activity using NIRS. In the study by Hada et al., they found that rTMS in healthy individuals had an intensity-dependent increase in [oxy-Hb] (30). Our findings are similar to those of Kawabata et al.’s study (31), where they reported increased frontal lobe activity measured by 15-channel NIRS in the frontal cortex of 15 patients with MDD after receiving 30 sessions of rTMS treatment; among them, 14 patients demonstrated significantly reductions in HAMD scores. However, the association between increased frontal lobe activity and symptom improvement was not reported in their study. In another study conducted by Huang et al. (32), they used low frequency rTMS on the right DLPFC to treat MDD patients. They also found increased frontal lobe activity after 20 sessions of treatment. However, they did not find any correlations between changes in frontal lobe activity and depressive symptoms. However, due to that HF-rTMS to the left DLPFC and LF-rTMS to the right DLPFC have different antidepressant effects on the brain (33), it is difficult to directly compare their study with ours.

Another finding in our study was that among rTMS responders, increased frontal lobe activity during VFT after 10 sessions of rTMS was associated with improved depressive symptomatology after 20 sessions of treatment, which has not been previously reported but is consistent with the findings reported by Yamagata et al. They found a significant inverse correlation between increased frontal lobe activity at 4 weeks and changes in HAMD score from 4 to 8 weeks and from 4 to 12 weeks in 11 medication-naïve patients with MDD who responded to sertraline treatment. They concluded that the biological marker could be used to predict the clinical response to treatment in MDD.

However, this study still has several limitations that should be addressed for future improvement. Firstly, this research was conducted in a naturalistic setting. A sham rTMS group was not used to control for placebo effects. Additionally, the sample size of this study was small and it did not include a control group (concurrent medication and psychotherapy groups, respectively). Moreover, small sample sizes may not provide enough statistic power to detect baseline frontal activity between responders and non-responders. These limitations should be considered when interpreting the findings, and future studies with larger sample sizes and appropriate control groups are needed to further investigate the effects of rTMS treatment in MDD.

## 5. Conclusion

In summary, our preliminary findings suggest that NIRS could serve as a potential biomarker for monitoring treatment responses to rTMS in patients with MDD. However, further studies with larger sample sizes are needed to confirm and validate our findings.

## Data availability statement

The original contributions presented in the study are included in the article/supplementary material, further inquiries can be directed to the corresponding authors.

## Ethics statement

The studies involving humans were approved by the Institutional Review Board of the Taichung Veterans General Hospital. The studies were conducted in accordance with the local legislation and institutional requirements. The participants provided their written informed consent to participate in this study.

## Author contributions

P-HC: conceptualization. P-HC and Y-LC: methodology. P-HC: formal analysis. W-HL: investigation. P-HC: writing—original draft preparation. P-HC, W-CL, S-CW, and C-HC: writing—review and editing. K-PS: supervision. P-HC: project administration. P-HC and K-PS: funding acquisition. All authors have read and agreed to the published version of the manuscript.

## Funding

This research was funded by National Science and Technology Council, An-Nan Hospital, China Medical University, Higher Education Sprout Project by the Ministry of Education (MOE), China Medical University, and China Medical University Hospital, Taiwan and this work was supported by the following grants: MOST 109-2320-B-038-057-MY3, 110-2321-B-006-004, 110-2811-B-039-507, 110-2320-B-039-048-MY2, 110-2320-B-039-047-MY3, 110-2813-C-039-327-B, 110-2314-B-039-029-MY3, 111-2321-B-006-008, and NSTC 111-2314-B-039-041-MY3 from the National Science and Technology Council, Taiwan; ANHRF 109-31, 109-40, 110-13, 110-26, 110-44, 110-45, 111-27, 111-28, 111-47, 111-48, and 111-52 from An-Nan Hospital, China Medical University, Tainan, Taiwan; CMRC-CMA-2 from Higher Education Sprout Project by the Ministry of Education (MOE), Taiwan; CMU 110-AWARD-02, 110-N-17, 1110-SR-73 from the China Medical University, Taichung, Taiwan; and DMR-106-101, 106-227, 109-102, 109-244, 110-124, 111-245, 112-097, 112-086, 112-109, 112-232 and DMR-HHC-109-11, HHC-109-12, HHC-110-10, and HHC-111-8 from the China Medical University Hospital, Taichung, Taiwan.

## Conflict of interest

The authors declare that the research was conducted in the absence of any commercial or financial relationships that could be construed as a potential conflict of interest.

## Publisher’s note

All claims expressed in this article are solely those of the authors and do not necessarily represent those of their affiliated organizations, or those of the publisher, the editors and the reviewers. Any product that may be evaluated in this article, or claim that may be made by its manufacturer, is not guaranteed or endorsed by the publisher.
